# Evaluating a Brief, Internet-Based Intervention for Co-Occurring Depression and Problematic Alcohol Use in Young People: Protocol for a Randomized Controlled Trial

**DOI:** 10.2196/resprot.3192

**Published:** 2014-02-27

**Authors:** Mark Deady, Maree Teesson, Frances Kay-Lambkin, Katherine L Mills

**Affiliations:** ^1^National Drug and Alcohol Research CentreUniversity of New South Wales (UNSW)SydneyAustralia

**Keywords:** depression, alcohol, young people, Internet-based, comorbidity

## Abstract

**Background:**

Depression and alcohol misuse represent two of the major causes of disease burden in young adults. These conditions frequently co-occur and this co-occurrence is associated with increased risks and poorer outcomes than either disorder in isolation. Integrated treatments have been shown to be effective, however, there remains a significant gap between those in need of treatment and those receiving it, particularly in young people. The increased availability of Internet-based programs to complement health care presents a unique opportunity in the treatment of these conditions.

**Objective:**

The objective of our study was to evaluate whether a brief, Internet-based, self-help intervention (the DEAL [DEpression-ALcohol] Project) can be effective in treating co-occurring depression and problematic alcohol use in young people (18-25 years old).

**Methods:**

The evaluation will take the form of a randomized controlled trial (RCT), comparing the DEAL Project with an attention-control condition (HealthWatch). The RCT will consist of a four-week intervention phase and a 24-week follow-up. It will be entirely Internet-based and open Australia-wide to young people 18 to 25 years old. The primary outcomes will be change in depression symptoms and alcohol use at 5, 12, and 24 weeks post baseline. Secondary outcomes include change in general functioning and quality of life, anxiety/stress symptomatology, and a number of other depression/alcohol related outcomes. Process analysis will also measure engagement across the conditions.

**Results:**

This study is currently ongoing with preliminary results expected in late 2014.

**Conclusions:**

This study, to our knowledge, will be the first RCT of a Internet-based treatment for comorbid depression and problematic alcohol use in any age group. If successful, the program represents a novel and innovative approach to addressing the significant harms associated with these conditions and will be an invaluable resource to those not receiving help elsewhere.

**Trial Registration:**

Australian New Zealand Clinical Trials Registry; ACTRN12613000033741; https://www.anzctr.org.au/Trial/Registration/TrialReview.aspx?id=363461 (Archived by WebCite at http://www.webcitation.org/6Mrg9VFX4).

## Introduction

### Alcohol Use Disorders and Depression

In the developed world, two of the top five leading global causes of years lost to disability are alcohol use disorders (AUD) and depression [1]. Young people bear a disproportionately large share of these burdens [2,3]. In Australia, the highest prevalence of 12-month AUDs in any age bracket occurs in those 16-24 years old (11.1%) [4]. In addition, this age bracket has the highest incidence rates for depression of any age group [5], half of whom will experience recurrent episodes of depression [6].

### Comorbidity

Comorbidity across the disorder classes is common. Approximately one in four (22.2%) young Australians (16-25 years old) with current major depression also meet criteria for a 12-month AUD, while 14.3% of those with a 12-month AUD meet criteria for current major depression [7]. In treatment samples, rates of comorbidity are as high as 89% [8].

These comorbid conditions are associated with increased suicidality [9-11], symptom severity, and poorer social, interpersonal, and general functioning compared to those with a single disorder [12-14]. This group is also likely to report poorer quality of life [15] and increased treatment reliance [16-21]. Furthermore, these disorders tend to maintain and exacerbate one another [22]. As such, in recent years there has been increasing support for integrated approaches to comorbidity [23,24]. Baker et al [25] have demonstrated that concurrent treatment of depression and problem drinking is more effective than treating either condition alone and more effective than general counseling.

Hides et al [26], suggest that treatment integration is particularly relevant to youth, given “coping” is a key motive for substance use among young people with mental health issues. There have, however, been few such attempts made in younger populations [27-29]. Although early intervention is imperative to averting the development of more severe, ingrained morbidity [30,31], fewer than 25% of affected young people access traditional health services in a 12-month period [32].

### Internet-Based Interventions

Internet-based interventions have been deemed to be particularly useful for those less likely to access traditional services, such as young people [33]. Advantages of these interventions include flexibility, anonymity, and accessibility, and as such, have the potential to overcome a number of structural and attitudinal barriers that frequently limit help seeking efforts in this population [34,35]. Furthermore, Internet use is widespread among young people [36,37]. Research suggests that the Internet helps to empower young people [38], and that young people are comfortable accessing both general health information and seeking specific mental health treatment via this medium [39,40]. Additionally, Internet-based treatments have the potential to reduce costs associated with treatment (by reducing contact time with the therapist), and increase treatment standardization and adherence to evidence-based practice [41,42]. Finally, this modality has also been shown to overcome imbalances in treatment seeking, access, and availability [43,44].

Such interventions have been shown to be effective and cost effective in treatment for depression and related disorders [45,46]. A number of recent meta-analyses have indicated that effect sizes for such interventions are small to moderate (0.28-0.78) [47-50], but roughly equivalent to traditional face-to-face therapy [51,52]. Similarly, recent meta-analyses have indicated that effect sizes for such alcohol interventions are small to moderate (0.22-0.48) [33,48, 53-55], again not dissimilar to brief in-person interventions [56,57].

To our knowledge, there has been no youth-focused Internet-based comorbidity interventions. Furthermore, in the general population only one computerized comorbidity intervention targeting these disorders has been evaluated. These evaluations of the computerized Self Help for Alcohol/other drug use and Depression (SHADE) resource indicate electronic forms of treatment for co-occurring disorders are viable and effective [58,59]. In two randomized controlled trials (RCTs), the SHADE program was associated with equivalent outcomes to that achieved by therapist-delivered treatment, with superior results as far as reducing alcohol consumption over 3- and 12-months.

Although unguided Internet-based interventions may not always be as effective as a face-to-face encounter with a skilled clinician, the reality is that a majority of those with depression and alcohol problems (especially young people) will never receive any face-to-face intervention. Fewer still will see a skilled clinician [32,60]. Internet-based interventions have the potential to engage young people through the use of new, interactive technology and may overcome the stigma associated with seeing a therapist.

### Objective

The primary aim of the study is to evaluate whether a brief, Internet-based intervention—the DEpression-ALcohol Project (DEAL)—can be effective in treating co-occurring moderate depression and problematic alcohol use in young people (18 to 25 years old). This evaluation will take the form of a RCT comparing the DEAL Project to an attention-control condition and measuring participant outcomes across time. This will be, to our knowledge, the first RCT of an Internet-based treatment for comorbid depression and problematic alcohol use in any age group.

## Methods

### Study Setting

The study will be conducted Australia-wide and entirely Internet-based with minimal participant contact. All contact made will occur via email, with the exception of follow-up contact from a clinical psychologist if participants report experiencing particular distress and are suicidal (see Safety Protocol).

### Study Design

**Figure 1:**
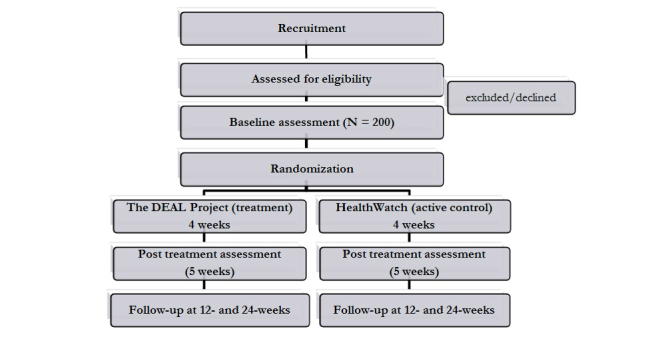
Flow of participants through the study.

The proposed RCT meets international standards for such trials. Figure 1 shows the design of the study and intended flow of participants. Initial contact with potential participants will be made via the Internet. Upon visiting the website, potential participants will complete an initial screening to determine eligibility. Eligible participants will receive a follow-up email to complete the full Internet-based baseline assessment, with randomization to one of the two treatment groups following assessment. Following randomization, participants will be provided with their login access code along with instructions about how to access the treatment website. Follow-up Internet-based assessment will occur across the two conditions at 5, 12, 24 weeks post baseline.

### Ethical Approval and Registration

The study is registered with the Australian New Zealand Clinical Trials Registry (ACTRN12613000033741) and has ethical approval from the University of New South Wales Human Research Ethics Committee (HC12546). Consent will be obtained electronically from all participants and confidentially assured via rigorous data encryption.

### Participants

We will aim to recruit approximately 200 participants. The trial has a purposive sample of young people reporting drinking at harmful levels with mild to moderate depressive symptoms. Young people will be informed that the study compares an Internet-based self-help program for depression and alcohol use with a healthy lifestyles program on a range of outcomes, and they will be randomly allocated to one of these groups. Recruitment will be Australia-wide, using extensive media coverage; including tertiary institutions and street press, Internet-based chat forums and blogs, government, youth-oriented services and websites, and paid Facebook and Google advertisements. The research team has been successful using similar strategies in previous studies [61]. They also have extensive experience recruiting large samples of participants with depression and alcohol use comorbidity (eg, 558 in 24 months in two states) [25,59].

### Inclusion and Exclusion Criteria

Inclusion criteria include: (1) 18-25 years old, (2) current depressive symptoms (score of ≥7 on Depression Anxiety Stress Scale-21-DASS-21-Depression) [62], (3) harmful drinking as measured by the Alcohol Use Disorders Identification Test (AUDIT) (score of ≥8) [63,64], (4) ability to access the Internet (either in the private residence of the participant, or willingness to use the public library/other suitable venue with Internet access), and (5) Australian residency.

Exclusion criteria include: (1) psychotic symptoms screener score ≥3 [65], (2) non-English speakers, (3) serious risk of suicide (serious past 2-week thoughts of suicide and desire to act), and (4) daily use of cannabis/weekly use of amphetamines.

### Randomization

Randomization will be automated within the program and therefore trial researchers will be blind to it. This process will occur immediately after the eligibility screener and baseline assessment have been completed and consent provided.

### Safety Protocol

In any trial concerned with mental health or substance use there is the potential to uncover psychological distress in participants. In this population there is an increased risk, as the participants are mild-moderately depressed and drinking at harmful levels. As recruitment (and the trial itself) is entirely Internet-based, no contact (other than email) will occur between participants and the research team during the course of the study. In order to minimize risk, study participants will be provided with a contact email of a clinician upon commencement of the trial. A trained clinical psychologist and member of the research team will monitor this email. Upon email contact, the psychologist will make contact with the participant and initiate a dialogue and negotiate local referral options where appropriate.

Upon weekly login, participants will answer a screening question regarding suicidality (thoughts of suicide or a wish to commit suicide in the past week). On the basis of their answer, they are prompted to the email contact of the psychologist.

At the beginning and end of each periodic assessment, the participant will again be presented with this referral information for crisis care.

### Interventions

Access to the treatment website for each of the following conditions will be for a period of 10 weeks from the point of randomization.

The DEAL Project program is a 4-week psychological treatment delivered entirely via the Internet. Participants access the Internet-based sessions from their home computer (or other preferred port for Internet access). Participant-selected pseudonym usernames/emails serve as their login code for the website. Content of the sessions is based on cognitive behavior therapy (CBT) and motivational enhancement theory and was developed from the SHADE resource [59]. Major modifications to the SHADE resource included length, language, reduction of text content, modified design and flow of program, and incorporation of youth vignettes. Participants are asked to complete each session of the DEAL Project intervention in sequence, a week apart, from the point of randomization. The website tracks participants’ progress through each weekly module, with automated email reminders sent to the participant’s nominated email address. However, participants are not “locked out” between sessions so they may complete at a faster rate.

There were 4 sessions of the HealthWatch program that were chosen for this study. This is an attention-control condition program first developed for the Australian National University WellBeing Study [66] in which participants read information about various health concerns and complete accompanying surveys. The purpose of this condition is to control for time spent interacting with an Internet-based program. The specific modules and surveys selected for the four weeks (from the complete 12-week set) were those deemed to be more relevant to younger people and include environmental health, physical and mental activity, nutrition, and relationships. These were redesigned to match the DEAL Project in appearance. Preliminary evidence from the WellBeing research trial suggests that the site is not associated with a reduction in depressive symptoms over time in adults.

### Assessment and Outcome Measures

All assessment tools are frequently used in mental health and alcohol research and in Internet-based (nonface-to-face) formats. Table 1 shows the schedule of assessments.

**Table 1:** Intended assessments and administration frequency.

Assessment instruments		Baseline assessment	Endpoint self-assessment (5 wks post baseline)	Follow-up self-assessments (12, 24 wks post baseline)
Demographics		✓	-	-
Service utilization		✓	-	-
**Depression**				
	Patient Health Questionnaire (PHQ-9) [67]	✓	✓	✓
	DASS-21 [62]	✓	✓	✓
**Alcohol**				
	AUDIT [63]	✓	✓^a^	✓^a^
	TOT-AL [68]	✓	✓	✓
	World Health Organization (WHO) Composite International Diagnostic Interview (CIDI-Alcohol) [69]	✓	✓	✓
**Other**				
	Opiate Treatment Index (OTI-other drugs) [70]^b^	✓	✓	✓
	Assessment of Quality of Life (AQoL) [71]	✓	✓	✓
	McLean Screening Instrument for Borderline Personality Disorder (MSI-BPD) [72]	✓	-	-
Program feedback		-	✓	-

^a^AUDIT-consumption items only

^b^modified to collect only general drug use data

### Administration of Assessments

Automatic email prompts to complete Internet-based follow-up assessments will be sent to participants at baseline, 5, 12, and 24 weeks post baseline. In line with best practice standards and our previous trial experience, the following strategies are employed to maximize retention in treatment and assessment: (1) email address required at program commencement, (2) individual email will be sent for each separate module, (3) reminder emails will be sent if participant does not complete assessment in six days, and (4) $10 iTunes voucher reimbursement provided for each scheduled assessments.

### Primary Hypotheses

It is hypothesized that integrated treatment for depression and alcohol problems (the DEAL Project) can achieve greater reductions in: (1) depressive symptoms, and (2) alcohol use, compared to an attention-control condition at 5, 12, and 24 weeks post baseline.

Secondary aims include the examination of: (1) general functioning and quality of life, (2) depression/anxiety/stress symptomatology, (3) hazardous alcohol use, (4) AUD criteria, and (5) engagement across the conditions.

### Primary Outcomes

Depressed mood is measured by the PHQ-9 [67], and alcohol use quantity and frequency is measured by the TOT-AL [68]. Prior experience with the target population suggests that both the treatment and attention-control groups are likely to show a decrease in these primary outcomes. We will also calculate reliable change indices (RCI) for depression and alcohol use at each time point relative to baseline to detect reliably significant change in primary outcome measures. RCI’s will be calculated using the methodology outlined in Jacobson and Truax [73], that is-(Ss_post_ – Ss_pre_)/SE_diff_ . Participants with an RCI of 1.96 or greater and who no longer meet the entry criteria for depression or hazardous/harmful alcohol use will be considered to have produced clinically and reliably significant change in these primary outcomes.

### Secondary Outcomes

In this study, the AQoL measures general functioning and quality of life [71], and depression/anxiety/stress symptomatology is measured by the DASS [62]. Hazardous alcohol use is measured by the AUDIT [63], and AUD criteria are measured by the CIDI-alcohol [69]. Engagement will be measured by a process analysis using website visit data.

### Data Analysis

As mentioned prior, the DEAL Project is based on the SHADE resource. Data from the SHADE trials indicate the program is associated with a 1.53 effect size change for depression and 0.86 for alcohol between baseline and 12-month follow-up assessments. However, given the SHADE intervention is therapist-guided and longer than that of the DEAL Project, we anticipate smaller effect sizes. Therefore, in line with other brief Internet-based alcohol multi-session modularized interventions with effect sizes of 0.56 [33], a more conservative medium effect size of 0.50 was used. These effects sizes were entered into GPower 3.1 [74] in order to estimate the sample sizes required to detect similar differences between the treatment and control conditions in the current study. Power was set at 80% and conservative 2-tailed tests were assumed even though a directional hypothesis is proposed. Based on this testing, a sample size of 64 per group was needed (total N=128). A conservative dropout rate is 35% of participants at follow-up. As such, we will aim to recruit 200 participants to the study. This will ensure that we will have sufficient power to conduct the analysis.

The authors, using available software packages, will carry out data coding and analysis. Data on screening, refusals, and dropout are coded and reported as per Consolidated Standards of Reporting Trials (CONSORT) [75], and primary analyses use intention-to-treat. Preliminary analyses check for any baseline or health service utilization differences that may confound with condition effects; later analyses control for these as necessary. Categorical and continuous measures of outcome will be examined using mixed or marginal longitudinal models (ie, mixed model repeated measures, generalized estimating equation modelling) as appropriate. These approaches enable the inclusion of participants with missing data, without using inferior techniques such as last observation carried forward, when data is missing at random [76]. A “completers” analysis on all participants completing at least 75% of the modules will be undertaken as a secondary analysis. In addition, comparisons on selected demographic and clinical characteristics will be made between “completers” and those who dropped out of treatment to help detect any biases in outcome measures. The potential effects of a number of covariates and confounders will be modelled in the major analyses (eg, borderline symptoms-MSI-BPD, medication status, drug use-modified OTI, gender, and involvement in additional mental health treatments) [72] [70].

## Results

Recruitment is currently underway with preliminary results expected in late 2014.

## Discussion

### The Present Study

The present study will assess the effectiveness of an Internet-based comorbidity intervention for young people. It is expected that depression and alcohol use outcomes for participants who complete the DEAL Project program will be significantly better than for those allocated to the control condition.

### Strengths and Limitations

A significant strength of the project is that it will be entirely Internet-based, without clinical guidance, thus, amplifying “real-world” applicability. This provides evidence on the feasibility of the intervention as a freely accessible program.

Previous trials of the SHADE program have used a guided approach [58,59], whereby therapists provide a one-session intervention at the commencement of treatment, along with 10-minute “check in” sessions at the conclusion of each computer session. This kind of approach has a number of advantages regarding therapeutic alliance, reduced dropout, improved utilization, and the ability to clarify concepts [77]. Unfortunately, such a technique is less likely to reflect real-world conditions.

A further strength of the proposed project is that the research design includes an active attention-control condition. The HealthWatch program will be used as the control, with participants being provided with a variety of health-related information. This active control [66] addresses limitations with previous clinical trials in which comparisons are allocated to a waitlist (no-treatment) control condition [78].

A potential challenge for this project will be participant dropout. Dropout rates from alcohol and other substance abuse treatment interventions are often high [79], similarly, Internet-based interventions, especially among young people, are also likely to compound these attrition rates [80]. It has been argued that a number of the strengths associated with Internet-based interventions (such as flexibility and anonymity) can quickly become weaknesses, as it may be much easier to neglect an appointment with an Internet-based program than a psychotherapist [81].

In a recent systematic review, Melville et al [82] found dropout rates from all Internet-based treatment programs for psychological disorders, which involved minimal therapist contact over a twenty year period, ranged from 2% to as high as 83%, with a weighted average of 31%. Interestingly, however, this weighted average was identical to that observed in face-to-face treatment for pathological gambling in the same review [82]. Therefore, assumptions that Internet-based therapies will automatically be associated with poorer adherence than face-to-face treatments appear contentious. Furthermore, the reasons for selective attrition are difficult to interpret as they may reflect the contradictory possibilities of dropout due to dissatisfaction, as opposed to dropout due to the individual feeling their needs have been met [83]. Nevertheless, attrition rates are a concern for any form of treatment and consequently the expansion of technology-based therapies demands researchers and developers consider innovative ways to engage individuals in therapy, particularly in younger populations. Feedback on the program will add to the knowledge base and aid future work in this area.

A related challenge is participant follow-up. Attempts to improve follow-up rates in the current study will include using a range of reminders, flexibility around timing of follow-up assessment (as it is Internet-based), obtaining a variety of contact details of significant others to help with locating participants, reinforcing to participants the importance of conducting follow-up, and financially compensating participants for the time required to complete the assessments ($10 vouchers for full completion of each assessment battery).

A final limitation is the program length. The program is considerably shorter than other interventions of this kind. However, brief interventions have been associated with significant effects for hazardous alcohol use outcomes compared to a variety of passive and active control conditions [84], especially among young populations [85]. A recent meta-analysis of 14 RCTs examined the effects of single-session personalized-feedback without therapeutic guidance on the reduction of problematic alcohol consumption in young adults [86]. The authors concluded such interventions were efficacious and cost effective and recommended the use of Internet-based approaches. This reiterated conclusions of an earlier review, which claimed that evidence supported the use of interventions that incorporated personalized feedback, either with or without practitioner support [87]. Similarly, in a systematic review of 22 RCTs of social norms-based brief interventions, Moreira et al [88] concluded that both computerized and individual face-to-face sessions appeared to reduce alcohol misuse.

Stice et al [89] reported that a four-week, group CBT-based intervention for adolescents at high-risk of depression was associated with significantly greater reductions in depressive symptoms, and a lower risk of developing depression, compared to bibliotherapy at 1- and 2-year follow-up. Scott et al [90] found primary care patients with depression who received six brief CBT sessions in combination with written educational material, recovered at significantly higher rates than those in standard care. These gains were maintained at 1-year follow-up. Similarly, a brief (4-6 sessions), solution-focused CBT treatment for depression was associated with a significantly greater reduction in symptom severity compared with standard care [91]. Brief (single session) interventions have also been found to be associated with comparable depression outcomes when compared to 10-sessions in samples with co-occurring alcohol problems [25].

Finally, in both depression and alcohol use interventions, young populations tend to be less likely to commit to the full course of sessions [92,93] and thus—especially in mild to moderate severity populations—it makes intuitive sense to adopt a brief-intervention approach.

### Conclusions

Problematic alcohol use and depression are significant problems facing young people today, however, a lack of service utilization in combination with a lack of specialized treatments mean most affected young people do not receive treatment. Internet-based interventions have the potential to overcome many of the barriers to treatment in this population. This will be the first RCT, to our knowledge, of a psychological therapy in young people with co-occurring alcohol and depressive problems [27]. The proposed trial focuses on a common clinical problem that causes substantial functional, economic, and health impacts-comorbid depression and problematic alcohol use in young people. These conditions are currently undertreated, contribute significantly to the global disease burden, and are at their peak in this age range. Offering treatments of low cost and with wide reach to affected people will address current inequities of treatment access for these problems and provide a youth-appropriate modality of treatment delivery. These results will have implications for service design and health policy, and speak to important questions about the nature of treatment effects in general. In particular, the study is in line with current national and international initiatives in eHealth and addresses important questions with both clinical and scientific significance.

## Abbreviations

AQoL: assessment of quality of life
AUD: alcohol use disorders
AUDIT: Alcohol Use Disorders Identification Test
CBT: cognitive behavior therapy
CIDI: Composite International Diagnostic Interview
CONSORT: Consolidated Standards of Reporting Trials
DASS-21: Depression Anxiety Stress Scale-21
DEAL: DEpression-ALcohol Project
MSI-BPD: McLean screening instrument for borderline personality disorder
NDARC: National Drug and Alcohol Research Centre
NHMRC: National Health and Medical Research Council
OTI: Opiate Treatment Index
PHQ-9: Patient Health Questionnaire-9
RCI: reliable change indices
RCTs: randomized controlled trials
SHADE: self help for alcohol/other drug use and depression
WHO: World Health Organization
